# How did extinct giant birds and pterosaurs fly? A comprehensive modeling approach to evaluate soaring performance

**DOI:** 10.1093/pnasnexus/pgac023

**Published:** 2022-03-10

**Authors:** Yusuke Goto, Ken Yoda, Henri Weimerskirch, Katsufumi Sato

**Affiliations:** Centre d'Etudes Biologiques de Chizé, CNRS, 79360 Villiers En Bois, France; Graduate School of Environmental Studies, Nagoya University, Furo, Chikusa, Nagoya 464-8601, Japan; Graduate School of Environmental Studies, Nagoya University, Furo, Chikusa, Nagoya 464-8601, Japan; Centre d'Etudes Biologiques de Chizé, CNRS, 79360 Villiers En Bois, France; Atmosphere and Ocean Research Institute, The University of Tokyo, Kashiwanoha, Kashiwa, Chiba, 277-8564, Japan

**Keywords:** birds, pterosaurs, wind, dynamic soaring, thermal soaring

## Abstract

The largest extinct volant birds (*Pelagornis sandersi* and *Argentavis magnificens*) and pterosaurs (*Pteranodon* and *Quetzalcoatlus*) are thought to have used wind-dependent soaring flight, similar to modern large birds. There are 2 types of soaring: thermal soaring, used by condors and frigatebirds, which involves the use of updrafts to ascend and then glide horizontally; and dynamic soaring, used by albatrosses, which involves the use of wind speed differences with height above the sea surface. Previous studies have suggested that *P. sandersi* used dynamic soaring, while *A. magnificens* and *Quetzalcoatlus* used thermal soaring. For *Pteranodon*, there is debate over whether they used dynamic or thermal soaring. However, the performance and wind speed requirements of dynamic and thermal soaring for these species have not yet been quantified comprehensively. We quantified these values using aerodynamic models and compared them with that of extant birds. For dynamic soaring, we quantified maximum travel speeds and maximum upwind speeds. For thermal soaring, we quantified the animal's sinking speed circling at a given radius and how far it could glide losing a given height. Our results confirmed those from previous studies that *A. magnificens* and *Pteranodon* used thermal soaring. Conversely, the results for *P. sandersi* and *Quetzalcoatlus* were contrary to those from previous studies. *P. sandersi* used thermal soaring, and *Quetzalcoatlus* had a poor ability both in dynamic and thermal soaring. Our results demonstrate the need for comprehensive assessments of performance and required wind conditions when estimating soaring styles of extinct flying species.

Significance Statement *Quetzalcoatlus* (10 m wingspan), one of the largest pterosaurs, was thought to have flown over land using updrafts like condors and eagles. Conversely, *Pelagornis sandersi* (7 m wingspan), one of the largest extinct volant birds, was thought to have flown using dynamic soaring like albatrosses, using differences in wind speed with height above the sea surface. In this study, we used aerodynamic models to comprehensively quantify soaring performances and wind requirements of these extinct species and compared them with extant soaring birds. We found that *Quetzalcoatlu*s was less suited to flying in updrafts than the extant birds, and *P. sandersi* was better suited to flying in updrafts above the sea, similar to frigatebirds, rather than using albatross-like dynamic soaring.

## Introduction

**Fig. 1.**
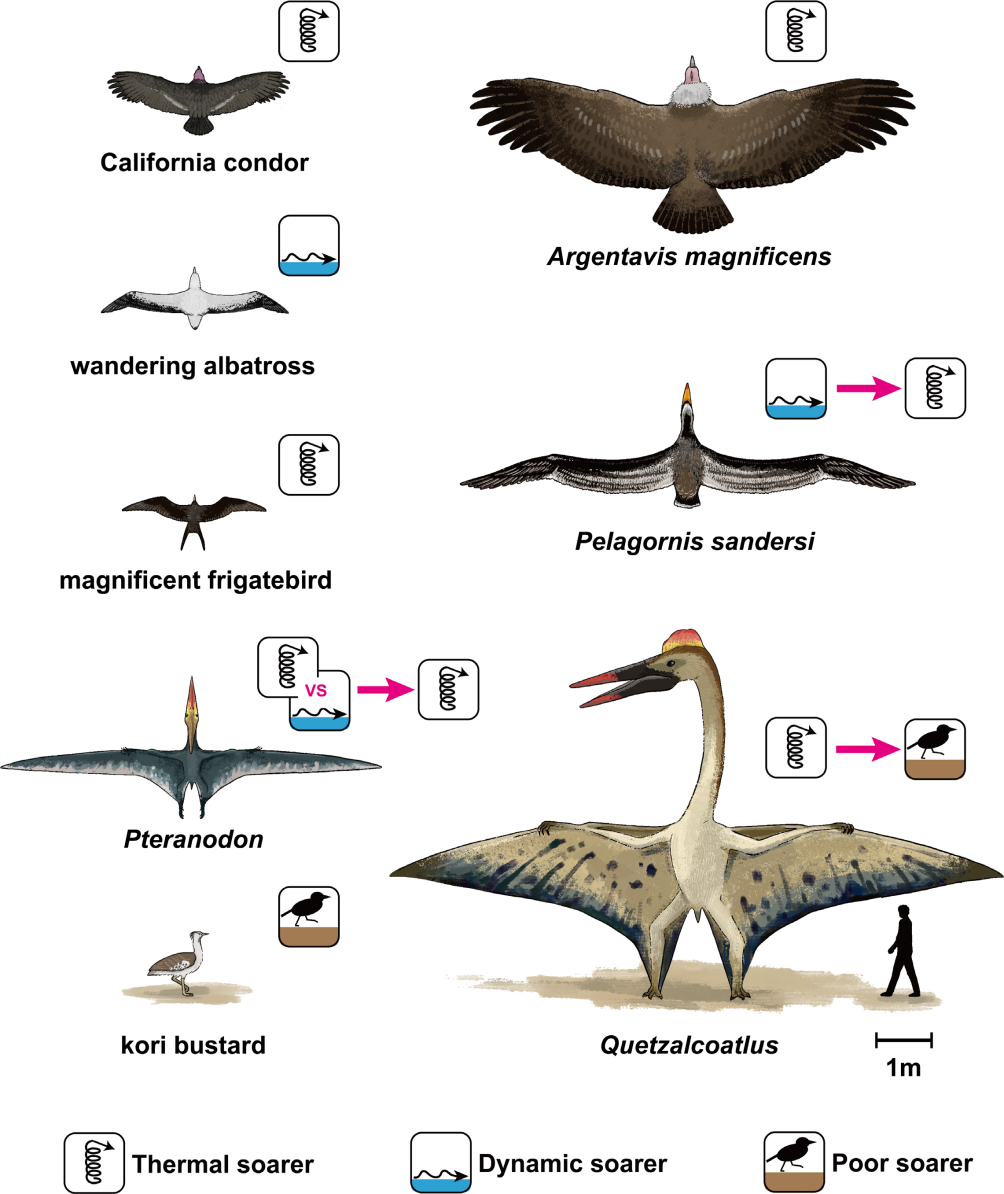
A size comparison and soaring styles of extinct giant birds (*P. sandersi* and *A. magnificens*), pterosaurs (*Pteranodon* and *Quetzalcoatlus*), the largest extant dynamic soaring bird (wandering albatross), the largest extant thermal soaring terrestrial bird (California condor), a large extant thermal soaring seabird (magnificent frigatebird), and the heaviest extant volant bird (kori bustard). The icons indicate dynamic soarer, thermal soarer, and poor soarer, and summarize the main results of this study. The pink arrows indicate the transition from a previous expectation or hypothesis to the knowledge updated in this study.

Flying animals have evolved a wide range of body sizes. Among them, there have been exceptionally large species of birds and pterosaurs (Fig. 1). Among the many extinct giant bird species (1–7), *Pelagornis sandersi* (from the late Oligocene, approximately 25–28 million years ago [Ma]) and *Argentavis magnificens* (from the upper Miocene, approximately 6 Ma) are the largest volant birds. Their estimated wingspans reached 6–7 m (1–4), twice as large as that of the wandering albatross [*Diomedea exulans*], the extant bird with the longest wingspan (Table 1). Several large species of pterosaurs appeared in the Cretaceous period. *Pteranodon*, arguably the most famous pterosaur, is estimated to have had a wingspan of 6–7 m (Table 1) (8, 9). The azhdarchids are one of the most successful Cretaceous pterosaur groups and include several large species with wingspans of approximately 9–11 m (Table 1) (10–13). Although their huge sizes have been led debate about whether they were flightless (14–16), *Quetzalcoatlus northorpi*, an azhdarchid species, is often regarded as one of the largest flying animals in history (16).

**Table 1:** Morphological values of examined species.

	Species	Mass [kg]	Wingspan [m]	Wing area [m^2^]	Aspect ratio	Wing loading [N/m^2^]	Ref
Extinct animals	*Pelagornis sandersi*	21.8 and 40.1	6.06, 6.13, 6.40, and 7.38	2.45–4.19	13.0, 14.0, and 15.0	51.0–87.4^a^ and 93.9–161^b^	(1)
	*Argentavis magnificens*	70.0	7.00	8.11	6.04	84.7	(4)
	*Pteranodon*	36.7	5.96	1.99	17.9	181	(9)
		18.6	5.34	2.26	12.6	80.7	(15)
	*Quetzalcoatlus*	259	9.64	11.4	8.18	224	(9)
Dynamic soaring birds	wandering albatross	8.64	3.05	0.606	15.4	140	(17)^c^
	black-browed albatross	3.55	2.25	0.376	13.4	87.5	(18)^c^
	white-chinned petrel	1.37	1.40	0.169	11.6	79.5	(19)
Thermal soaring birds	magnificent frigatebird	1.52	2.29	0.408	12.8	36.5	(20)
	California condor	9.50	2.74	1.32	5.70	70.6	(4)
	brown pelican	2.65	2.10	0.450	9.80	57.8	(20)
	black vulture	1.82	1.38	0.327	5.82	54.6	(20)
	white stork	3.40	2.18	0.540	7.42	61.8	(4)
Non-soaring bird	kori bustard	11.9	2.47	1.06	5.76	110	(21)^d^
Motor glider	Schleicher ASK 14	340	14.3	12.6	16.2	265	(22)

a With 21.8 kg mass.

b With 40.1 kg mass.

c We used the averages calculated from the morphological values of males and females in the cited references.

d We used the morphology data available in the *Flight* program.

How and how well these giant animals were able to fly has fascinated researchers across disciplines for over a century (23). This is because the question is not only interesting as a biophysical question in its own right, but also because it contributes to unraveling a wide range of issues such as the lifestyle of these species, their role in paleoecosystems, and the drivers of morphological evolution, diversification, and extinction of giant species over geological time (6, 16, 24–26). Their huge size must have significantly affected their flight because, with increasing size, the power required to fly increases faster than the power muscles can produce via the flapping of wings (27–29). Hence, this physical constraint has resulted in 2 debated arguments about the flight of extinct giants. The first debate is about whether and how they were able to take off (4, 14, 30, 31). The second debate concerns their soaring flight style. Due to the high costs of flapping that stems from their large body size, large extant birds prefer to fly utilizing wind energy or convection, that is, they prefer to soar (14, 32). Hence, it is presumed that extinct large animals also employed soaring flight as their primary mode of transportation (1, 4, 16). However, the kind of soaring flight style they employed is debated (1, 4, 16, 33, 34). The present study focuses on this second debate.

**Fig. 2.**
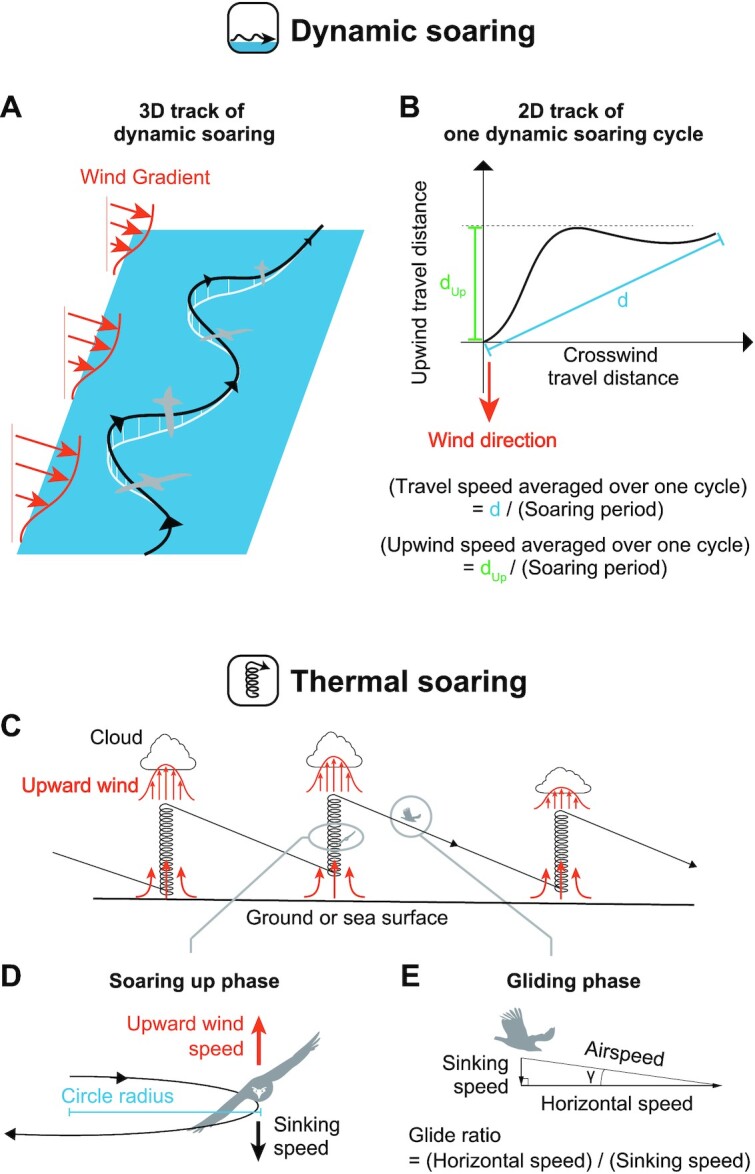
Schematics of dynamic soaring and thermal soaring. (A) Example of a 3D track of dynamic soaring. Dynamic soaring species repeat an up and down process with a shallow S-shaped trajectory at the sea surface. By utilizing wind gradients, a species can fly without flapping. (B) Example of a 2D dynamic soaring trajectory of 1 soaring cycle. The travel speed averaged over 1 cycle is defined as the travel distance in 1 cycle (d) divided by the soaring period, and the upwind speed averaged over 1 cycle is defined as the upwind travel distance in 1 cycle (d_Up_) divided by the soaring period. (C) Schematic of a thermal soaring cycle. (D) In the soaring up phase, a species soars in a steady circle. When there is upward wind that is greater than a species’ sinking speed, the species can ascend in the thermal. The upward wind is stronger in the center of a thermal; therefore, achieving a small circle radius is advantageous for thermal soaring. (E) In the gliding phase, a species glides in a straight line. The rate of horizontal speed to the sinking speed is equal to the rate of horizontal distance traveled to the height lost.

There are 2 main soaring flight styles among extant birds: dynamic soaring and thermal (and slope) soaring (21). In dynamic soaring, birds extract flight energy from wind shear—the vertical gradient in horizontal wind speed over the ocean (Fig. 2A–B). Extant seabirds (e.g. albatrosses, shearwaters, and petrels) employ this soaring style and can routinely travel hundreds of kilometers per day over the sea. In thermal soaring, birds first fly circling in warm rising-air columns (thermals). They climb to a substantial height and then glide off in the desired direction while losing their height (Fig. 2C–E). By repeating this up–down process, birds travel over vast distances. Various terrestrial bird species (e.g. vultures, eagles, and storks) and seabirds (e.g. frigatebirds and pelicans) employ thermal soaring (20). Terrestrial birds that utilize thermal soaring are also able to use the upward wind deflected by a mountain or hillside instead of the thermal for their ascent (32); this is termed “slope soaring.” Another form of slope soaring called wave-slope soaring uses the upward wind generated by waves (35). Wave-slope soaring is thought to be used by both thermal soaring seabirds (pelicans) and dynamic soaring seabirds (albatrosses) (35), but since no bird exclusively uses wave-slope soaring, we did not include it in the main soaring styles here.

Previous studies estimated that *P. sandersi* was a dynamic soarer (1), and *A. magnificens* and *Quetzalcoatlus* were thermal soarers (4, 16). *Pteranodon* was estimated to be a dynamic soarer (16), but subsequent studies have indicated that it was a thermal soarer (34, 36). See *Quantification of soaring styles in previous studies* in Materials and Methods for details about previous studies on this topic.

To estimate the potential soaring styles of these extinct animals, it is necessary to quantify their soaring performance, e.g. potential speed and efficiency of soaring, as well as the required wind speed to sustain soaring flight. Valuable indicators of dynamic soaring performance are the maximum travel speed and the maximum upwind speed averaged over 1 dynamic soaring cycle (Fig. 2B) (37–39). Additionally, it is necessary to evaluate the minimum horizontal wind speed required for sustainable dynamic soaring (40). Thermal (and slope) soaring performance is well-quantified by 2 indicators: the glide ratio, i.e. the ratio of the distance the animal traverses to the height the bird loses to cover that distance in the gliding phase (Fig. 2E), and the sinking speed of the animal circling in a given radius during the upward soaring phase (Fig. 2D). This sinking speed during circling corresponds to the upward wind speed required to ascend in a thermal. Because thermals have a stronger updraft in the center (Fig. 2C), the animal needs to achieve not only low sinking speed but also a narrow circle radius to efficiently ascend using a thermal. For slope soaring, the minimum value of sinking speed (sinking speed at infinite radius) would be an appropriate metric for performance, as the circle radius is less restrictive in slope soaring.

However, the soaring performances and required wind conditions have not been comprehensively evaluated for *P. sandersi, A. magnificens, Pteranodon*, and *Quetzalcoatlus* (summarized in Table 2). A total of 3 knowledge gaps are highlighted in Table 2: (1) the dynamic soaring performance and the minimum required wind speed have never been evaluated; (2) the thermal soaring performance in the soaring up phase and the required updraft wind speed have not been evaluated for *P. sandersi, Pteranodon*, and *Quetzalcoatlu*s; and (3) despite recent studies showing that the body masses of *Pteranodon* and *Quetzalcoatlus* were approximately 3 times heavier than previously expected (9, 14, 15), and that pterosaurs’ wings had a higher profile drag than that of birds (34), the soaring performances of these new heavy body masses and higher drags have rarely been evaluated.

**Table 2:** Summary of previous studies. Previous studies that quantified the soaring performances and required wind conditions of *P. sandersi, A. magnificens, Pteranodon*, and *Quetzalcoatlus* with recent heavy body mass estimates.

		**Dynamic soaring**		**Thermal soaring**		
**Species**	**Predicted soaring style**	Wind condition	Performance	Wind condition	Performance (Gliding)	Performance (Soaring up)
*Pelagornis sandersi*	Dynamic soaring (1)	—	Glide polar (1)	—	Glide polar (1)	—
*Argentavis magnificens*	Thermal soaring (4)	—	—	Circling envelope (4)	Glide polar (4)	Circling envelope (4)
*Pteranodon*	Thermal soaring (34) Dynamic soaring (16)^†^	—	—	—	Glide polar (34)	—
*Quetzalcoatlus*	Thermal soaring (16)^†^	—	—	—	—	—

† A principal component analysis (PCA) using 3 morphological information (logarithm of each of the animal's weight, wing area, and wingspan) has been performed in the previous studies for birds (41), bats (42), and pterosaurs (9), respectively. In the previous study (16), these second and third principal components were compared among birds, bats, and pterosaurs to estimate the soaring styles of pterosaurs.

In this study, we aimed to address these knowledge gaps and identify the potential soaring styles of these extinct giant birds and pterosaurs. To this end, we used physical models and recent morphology estimates to quantify the performance and wind conditions required for dynamic and thermal soaring in these extinct animals, and compared them with those of extant soaring birds. See *Models* in Materials and Methods for details about the employed models and parameter values.

Another aim of this study is to provide a framework for the objective evaluation of the soaring performance of extinct animals using models based on physical principles that all animals adhere to and serve as a stepping stone for invoking further research on this topic. Therefore, the assumptions and simplifications in our model, as well as our results and their implications, are important outcomes for future research and are explored in depth in the Discussion section.

## Results

### Dynamic soaring

**Fig. 3.**
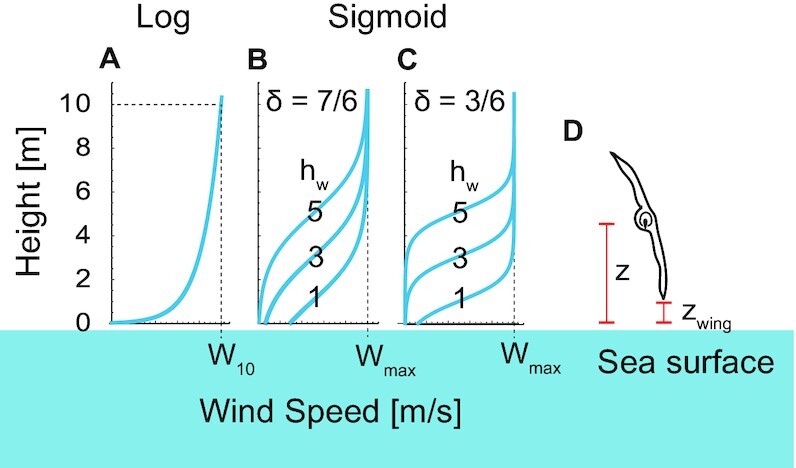
Wind shear models explored in this study. (A) Logarithmic wind gradient model. The wind speed at height 10 m was defined as *W*_10_. (B) Sigmoidal wind shear model with a wind shear thickness of 7 m (δ = 7/6) and a shear height (*h*_w_) of 1, 3, or 5 m. (C) Sigmoidal wind shear model with a wind shear thickness of 3 m (δ = 3/6) and a shear height of 1, 3, or 5 m. The maximum wind speed of the sigmoidal model is represented as W_max_. (D) Schematic of a soaring bird. Its height from the sea surface is represented as z and the height of the wingtip is represented as z_wing_. We constrained the models so that the wing tip did not touch the sea surface, i.e. *z*_wing_ ≥ 0.

We quantified the dynamic soaring performance (Fig. 2B) and required wind speeds using a physical model and a numerical optimization method. This method has been developed in the engineering field and provides a framework to quantify dynamic soaring performances and required wind conditions for gliders and birds (37, 40, 43, 44). However, despite its effectiveness, the only animal to which this technique has been applied is the wandering albatross (40, 44); it has never been applied to extinct giant flyers. We applied this framework to the 4 giant extinct species and 3 extant dynamic soaring bird species with various sizes ranging from 1 to 9 kg (i.e. the white-chinned petrel [*Procellaria aequinoctialis*], the black-browed albatross [*Thalassarche melanophris*], and the wandering albatross). As the exact shape of the wind gradient remains poorly understood, we conducted the calculation under 7 different wind conditions (Fig. 3A–C) (40, 44). In addition, we added an important modification to the previous models: the animal's wings do not touch the sea surface during their flight (Fig. 3D; and see Eq. 17 and its description in Materials and Methods for details).

**Fig. 4.**
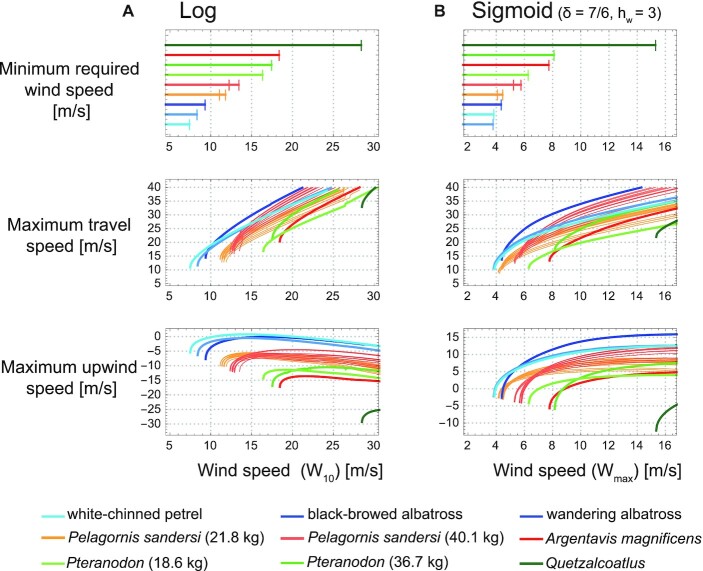
Minimum required wind speeds and dynamic soaring performances of extinct and extant animals. (A) Results of the logarithmic wind model. (B) Results of the sigmoidal wind model with a wind shear thickness of 7 m (δ = 7/6) and wind shear height (*h*_w_) of 3 m. The first row shows the minimum required wind speed for sustainable dynamic soaring. The second row shows the maximum travel speed averaged over 1 soaring cycle, in response to wind speed. The third row shows the maximum upwind speed averaged over 1 soaring cycle, in response to wind speed.

Our computation results indicate that *A. magnificens, Pteranodon*, and *Quetzalcoatlus* showed lower dynamic soaring performances and higher required wind speeds for dynamic soaring than the extant dynamic soaring species under all wind conditions tested in this study (Fig. 4; Figures S1 and S2, Supplementary Material). Therefore, these species could not have employed dynamic soaring.

For *P. sandersi*, the results were highly dependent on body mass. With heavy body mass estimates (40.1 kg), *P. sandersi* required higher wind speeds than extant dynamic soaring species, irrespective of the wind conditions. The performance was superior to extant species for some morphology estimates when the shear height was far from the sea surface (sigmoidal wind condition with *h*_w_ = 5 m),  but inferior when the wind speed change was located close to the sea surface (logarithmic model and sigmoidal wind condition with *h*_w_ = 1 and 3 m). In particular, their upwind speed was inferior to that of extant species, resulting in an inefficient flight at their upwind destination and, consequently, limiting their travel direction. When lower body mass estimates were used (21.8 kg), *P. sandersi* required lower wind speeds, but its performance was distinctively lower than that of extant species. Hence, *P. sandersi* required harsh wind conditions for dynamic soaring when a 40.1 kg body mass was assumed, and it was poor at dynamic soaring when a 21.8 kg body mass was assumed.

The performances of all species varied with the value of *h*_w_, and the variation was especially distinct for large species in contrast to that of white-chinned petrels (Fig. 4; Figures S1 and S2, Supplementary Material). This variation was due to wingtip boundary conditions (Fig. 3D and Eq. 17). Animals can attain more energy when passing through large wind speed gradients, but when large gradient changes are close to the sea level, large animals are unable to use the wind speed gradient efficiently because their wings limit the altitude available to them. Although the long, thin wings that reduce drag in extant dynamic soaring birds are suited for dynamic soaring (21, 45), our detailed dynamic models have shown that excessively long wings can also inhibit efficient dynamic soaring.

### Thermal soaring

The thermal soaring performances and the required upward wind speeds (Fig. 2D and E) were quantified using the established framework, i.e. glide polars and circling envelopes. A glide polar is a graphical plot of the sinking speed vs. horizontal speed when a bird glides in a straight line (21). We can determine the maximum glide ratio and the associated travel speed of flyers by identifying the line that passes through the origin and tangents of the glide polar plot. The inverse of the line slope and the speed at the tangent point correspond to the maximum glide ratio and the associated horizontal speed, respectively. The circling envelope is a graphical plot of the sinking speed (i.e. equivalent to the required upward wind speed for ascent in a thermal) vs. the radius of a turn when a bird glides in a steady circle (21, 20).

We quantified the thermal soaring performances and the required upward wind speeds for the 4 extinct species, 5 extant thermal soaring species (the magnificent frigatebird [*Fregata magnificens*], the black vulture [*Coragyps atratus*], the brown pelican [*Pelecanus occidentalis*], the white stork [*Ciconia ciconia*], and the California condor [*Gymnogyps californianus*]), and the kori bustard (*Ardeotis kori*), the heaviest extant volant bird that does not soar. The performances reported in a previous study of the Schleicher ASK-14, a motor glider with a 14 m wingspan similar to that of *Quetzalcoatlus*, are also presented for comparison (22).

**Fig. 5.**
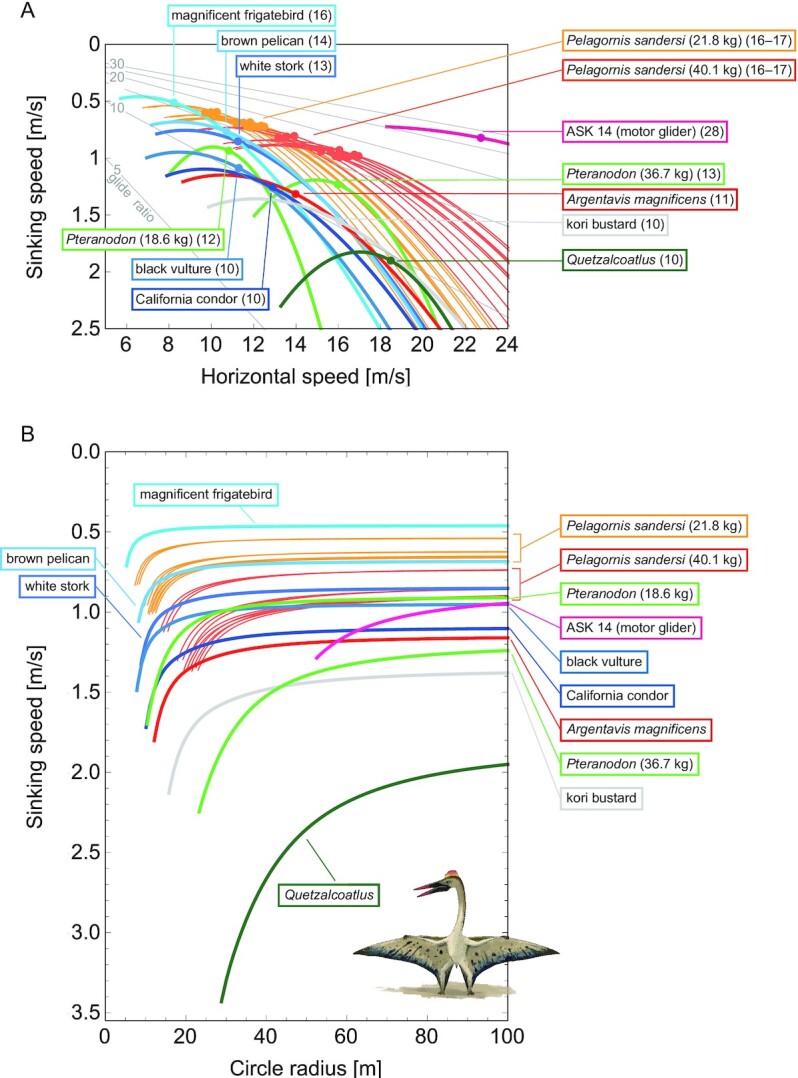
Glide polars (A) and circling envelopes (B) of extinct species, extant thermal soaring species, and the kori bustard, the heaviest rarely flying bird. In (A), the maximum glide ratios of each species are shown on the right side of species names. Points represent the horizontal speed and sinking speed at the maximum glide ratio of each species. Gray lines represent glide ratios (5, 10, 15, 20, 25, and 30). (B) Shows circling envelopes defined as the minimum sinking speed against the circle radius. The left end of the curve is for a bank angle of 40°. The smaller the bank angle, the larger the circle radius. A linear wingspan reduction is assumed for birds and a fixed wingspan is assumed for pterosaurs. The lift coefficient of circling envelope ($C_L^*$) is the maximum lift coefficient (1.8 for birds and 2.0 for pterosaurs).

All extinct species showed high gliding performances with maximum glide ratios ranging from 10 to 17 (Fig. 5A), which are comparative to those of extant species.

With respect to the soaring up phase, all of the extinct giant flyers, except for *Quetzalcoatlus*, had performances equivalent to or better than the extant soaring species (Fig. 5B). As shown previously (4), the circling up performance of *A. magnificens* was comparable to that of the California condor, one of the largest living thermal soarers. The performance of *Pteranodon* was comparable to that of living thermal soarers; however, it was rather low when the higher body mass estimate (36.7 kg) was employed. The thermal soaring ability of *P. sandersi* when a light mass was assumed (21.9 kg) was outstanding. It outperformed several extant thermal soaring species in soaring up ability, and was even comparable to the magnificent frigatebirds, the champion of thermal soaring among extant species. Even with a heavier body mass estimate (40.1 kg), *P. sandersi* still outperformed or was comparable to several other species.

Among the 4 extinct giant animals investigated in this study, the soaring up performance of *Quetzalcoatlus* was exceptionally low. It required the strongest upward wind speed and the widest circle radius. Its performance was even lower than that of the kori bustard, one of the heaviest volant extant bird species, which spends most of its time on land and only fly in emergencies, such as when under predation risk.

Figure 5 also demonstrates how gliders and animals differ with regards to what they prioritize in thermal soaring. A low sinking speed and a narrow circle radius are advantageous during the ascent phase of thermal soaring, whereas a high glide ratio and flight speed are advantageous during the glide phase, the benefits of which are not simultaneously satisfied. Natural selection has favored bird species that prioritize performance in the ascent phase; thermal soaring species have a smaller wing loading relative to their size than other species (45). This aids in achieving a slower sinking speed and tighter circle radius, as they must use weak updrafts at low altitudes after landing on the ground for foraging and/or flying in poor updraft conditions due to unexpected weather changes. Conversely, since the development of gliders in 1911, their designers have changed their performance priorities (46). In the early stages of glider development, the ability to climb in weak updrafts was prioritized, as is the case in birds, with gliders having large wing areas, low aspect ratios, and low wing loadings. Subsequently, performance in the glide phase was increasingly prioritized, leading to the development of gliders with high aspect ratio wings and high wing loading to achieve a high glide ratio and fast flight speed. In addition, as the high aspect ratio reduced the induced drag, this change was also advantageous in reducing the sinking speed. This change was possible through a combination of the discovery of flight strategies that use strong thermals at high altitudes, and the development of new high-strength materials, such as carbon-reinforced plastic, that allowed elongated wings to withstand high-speed flights (46). The performance of the motor glider (ASK 14) in Fig. 5 indicates that while its high flight speed results in a larger circle radius than that of animals, its maximum glide ratio is nearly 30, far superior to that of birds and pterosaurs. Some modern sailplanes (such as Eta) have glide ratios of up to 70 and aspect ratios up to 50 (47). Therefore, the distinct difference in performance between the glider and animals (Fig. 5) indicates the difference in “selection pressure” and constraints on morphology (i.e. materials available for the wing) between them.

## Discussion

Although several previous studies have investigated the soaring performance of extinct species, there have been several evaluation gaps. In the present study, we filled these gaps using physical models of soaring birds. We computed and compared the dynamic and thermal soaring performances and the required wind conditions for soaring of 4 extinct giant flyers with those of extant dynamic and thermal soaring species, which enabled us to examine the soaring style of extinct giants from multiple perspectives. Our results indicate that *A. magnificens* was a thermal soarer, confirming a previous study (4). For *Pteranodon*, there were 2 previous competing claims: dynamic soarer (16) and thermal soarer (34). Our results supported that they were thermal soarers. For *Quetzalcoatlus* and *P. sandersi*, our results were in contrast to previous studies. Our results indicate that *Quetzalcoatlus* could not efficiently perform dynamic nor thermal soaring. In addition, although *P. sandersi* was considered a dynamic soaring species in a previous study (1), our results suggest that it was a thermal soaring bird. We discuss our results in detail for *Pteranodon, Quetzalcoatlus*, and *P. sandersi*, and then describe future issues that need to be addressed for a better understanding of the soaring styles of extinct giant species.

### Pteranodon

There have been conflicting estimates of the soaring style of *Pteranodon*. It was claimed that they used thermal soaring based on quantitative evaluations of glide polars obtained from wind tunnel experiments using reconstructed wings of pterosaurs (34) and of the strength of the pterosaur's wing membranes using a physical model (36). In contrast, it was claimed that they used dynamic soaring based on a comparison of morphology between birds, bats, and pterosaurs with a principal component analysis (PCA), which showed that *Pteranodon* and albatrosses were located in similar regions of the principal components space (16).

However, it should be noted that the comparative PCA method employed in the latter study does not provide quantitative soaring performances, as cautioned by the proposer of the original method (41) (this issue will be discussed in detail in the next section, “*Quetzalcoatlus*”). Hence, there has been no direct evaluation of the dynamic soaring performance of *Pteranodon*. Our results fill this knowledge gap and show that *Pteranodon* was not good at dynamic soaring and had a thermal soaring ability comparable to that of extant species, as has already been shown (34).

Based on these points, and as many *Pteranodon* have been found from deposits in the center of the Western Interior Seaway (48), we suggest that they travelled over oceans by thermal soaring like frigatebirds.

### Quetzalcoatlus

There has been a heated debate about the flight capability of *Quetzalcoatlus*. The focal issue has been whether or not *Quetzalcoatlus* could take off. Researchers are divided between the opinion that it was too heavy to take off (14,15) and the opinion that it was able to take off by using quadrupedal launching, like some bats (16, 30) (but also see the recent discussion on the launching of *Quetzalcoatlus* (49)). In addition, detailed observations of fossils are also presented as evidence that the giant azhdarchids, including *Quetzalcoatlus*, were capable of flight; for example, a huge deltopectoral crest on their humeri, which would have anchored muscles for flapping flight (50).

Although there is some debate as to whether or not giant pterosaurs could have taken off, it has been widely accepted that if they were able to take off their primary mode of travel would have been thermal soaring rather than flapping flight (16). Witton and Habib reported that the flapping flight of *Quetzalcoatlus* required anaerobic movement and was difficult to sustain for a long period; therefore, it must have relied on wind energy for long-distance travel (16). Based on a comparison of morphology between birds, bats, and pterosaurs with a PCA, these authors also concluded that *Quetzalcoatlus* used thermal soaring (9, 16).

Our results revealed that *Quetzalcoatlus* had a poor performance to ascend using thermal or deflected upward wind. It required a larger circle radius and stronger updraft than the extant thermal soaring species, and even than the kori bustard that does not soar and spent most of their time on land. This suggests that the wind conditions under which *Quetzalcoatlus* could conduct sustainable thermal soaring were limited. The ability to climb in weak updrafts at low altitudes is particularly important for sustainable thermal soaring. At high altitudes, strong thermals can create updrafts of 5 m/s or more, and thus the sink rate of approximately 2 m/s of *Quetzalcoatlus* would have been sufficient to exploit such strong updrafts. However, it should be noted that thermals are generally weaker and narrower at low altitudes (51). Therefore, every time an animal starts to climb a thermal, they are obliged to soar in the poor thermal conditions at low altitudes. For example, the largest existing thermal soaring species can only climb at low speeds at low altitudes because of the weaker updrafts of thermals and the bird's high sink rate involved in reducing the circle radius to accommodate the narrower thermal width (51). Hence, the poorer thermal soaring ability of *Quetzalcoatlus* than that of extant species would have greatly reduced the number of thermals they could have climbed, which would have made it difficult for them to stay aloft long enough to find a strong thermal.

The poor thermal and slope soaring performance of *Quetzalcoatlus* was due to the large wing loading associated with the large body size. As shown in Materials and Methods, the circle radius was proportional to the wing loading (Eq. 21), and the minimum sinking speed during straight glide was proportional to the wing loading to the power of one-half, when the effect of the wing length adjustment and the lift coefficient dependence of the profile drag coefficient were negligible (Eq. 20). Since the wing loading increases with body mass, a giant *Quetzalcoatlus* required thermals with a wider radius and stronger updraft for thermal and slope soaring.

The wing loading also explains why the results of the present study are not consistent with the claims of previous studies that *Quetzalcoatlus* was adapted to thermal soaring (9, 16). In the previous study, soaring ability was assessed from 2 variables related to wing loading and aspect ratio with PCA (second and third principal components obtained by PCA on the logarithms of body weight, wing area, and wingspan; see (41, 42) for details of the method and data). Note that these variables are not exactly equal to wing loading and aspect ratio. In particular, the size-dependence has been removed from the principal component related to wing loading. However, thermal soaring performance is inevitably size-dependent and, therefore, using performance and wind requirements calculated from morphology based on the laws of physics (as conducted in this study and (26, 45)) are more accurate.

Interestingly, a recent research on *Quetzalcoatlus* suggested that their wing loading is even higher than previously estimated (49). The authors suggested narrower pterodactyloid wings, which are little or not attached to the hind limbs, while many previous studies adopted the wings that are attached to the hind limbs and have a broad, bat-like shape (16, 36, 52). Although no specific morphological estimates are provided in the study (49), if their argument is correct, *Quetzalcoatlus* would have had a smaller wing area, and thus even greater wing loading than previously estimated, resulting in less thermal soaring capability than that estimated in our study.

Anatomical studies of the azhdarchid pterosaurs have reported that their skeletal structure shows adaptations to terrestrial walking and suggested that they were terrestrial foragers (52, 53). Furthermore, a recent phylogenetic analysis showed that the azhdarchoid pterosaurs differed from other pterosaurs, in that they had evolved in a manner that increased the cost of transport for flapping flight and the sinking speed of gliding (26). Taking into account the adaptations for walking (52, 53), the humeri feature indicating flapping flight capability (50) but not sustainable flapping flight (16), the phylogenetic tendency of decreasing flight efficiency (26), and the low thermal soaring ability shown here, we suggest that the flight styles of *Quetzalcoatlus* and other similar-sized azhdarchid *s*pecies were similar to those of the bustard or ground hornbill that are short-range flyer and spend most of their time on land. Several previous studies overstated the flight ability of large azhdarchid pterosaurs such that they had a “capacity for nonstop flights exceeding 10,000 miles” (54) and an “unprecedented capacity for trans-continental flight" (Fig. 8 of (55)). However, our results indicate that these claims seem highly unlikely.

### Pelagornis sandersi

As *P. sandersi* was found close to the coast, this species is thought to have lived an oceanic existence by soaring over the sea (1). Previously, it has been reported that *P. sandersi* was a dynamic soarer like the albatross, rather than a thermal soarer like frigatebirds (1). However, we argue that this species is highly adapted for thermal soaring. The conclusion of the previous study was based on the glide polars of *P. sandersi*, which were more similar to those of the wandering albatrosses than those of frigatebirds; glide performance was the only criterion used to evaluate its soaring style. In this study, we quantified other performances and the required wind conditions, which enabled us to evaluate the soaring style of *P. sandersi* from multiple perspectives.

Our results indicated that the dynamic soaring performance of *P. sandersi* was generally inferior to that of extant dynamic soaring species, although there were substantial variations depending on wind conditions and morphology estimates. One of the factors contributing to the poor dynamic soaring ability of this species was an inability to efficiently exploit the wind speed gradient due to long wings limiting the height above sea level at which the bird could fly. This effect could not be assessed using the glide polars alone.

Conversely, the thermal soaring ability of *P. sandersi* was outstanding, regardless of the morphology estimates used. The thermal soaring ability of an animal is largely dependent on its wing loading, as previously discussed. Therefore, the reason why *P. sandersi* showed such a high performance is because of its low wing loading despite its huge size. Considering that *P. sandersi* was found close to the coast, this species is expected to have been well-adapted to capture weak updrafts above the sea by using thermal soaring, and that it was able to stay aloft for a long period of time with limited flapping and traveled long distances, similar to frigatebirds (56).

### Future issues

In this section, we discuss some of the simplifications used in this study and issues that we believe need to be addressed in the future. Below, we explore 3 issues with modeling flight: flight stability, wind environments, and discrepancy between model predictions and actual dynamic soaring speeds.

The first issue is flight stability. In this study, a steady wind environment was assumed, but actual wind environments fluctuate. In such a fluctuating real-world environment, stability is an important factor that determines the success or failure of flight (57–59). To simplify our calculations, we did not address stability, but it is important to examine the flight stability of these extinct and extant birds using more detailed morphological information in the future.

The second issue is that the actual wind environment experienced by both extinct and extant animals is still largely unknown.

For dynamic soaring, the specific form of the wind speed gradient experienced by birds is unknown—for example, whether there is a logarithmic or sigmoidal gust in the shadows of waves (44, 60). For this reason, we evaluated performance under various wind conditions (Fig. 3). For thermal soaring, it is also unknown how much updraft animals experience at a given circle radius or the distance between thermals. Recent advances in tracking technology have made it possible to record details of the motion of birds in dynamic and thermal soaring (51, 61–64). These data will provide information of the real wind environment experienced by soaring animals (64–68).

It is also important to consider the paleoenvironmental aspects of the wind environment at the time of the extinct species' inhabitation. For example, we showed that *Quetzalcoatlus* had a lower thermal soaring capacity than the extinct species. More detailed paleoclimatic estimates may help us to understand whether the species experienced a quite extreme wind environment to enable them to use thermal soaring as its primary mode of transport, even with their poor soaring up ability. As another example, we showed that *Pteranodon* and *Pelagornis sandershi* were oceanic thermal soarers like frigatebirds. Frigatebirds are distributed in trade wind zones with abundant updrafts (20,56); however, *Pteranodon* and *Pelagornis* had a wider distribution (1,53). These mismatches between extant and extinct oceanic thermal soaring species could reflect the difference in the climatic environment between the modern era and the era of the extinct giants.

Third, it should be noted that in our model of dynamic soaring, the maximum travel speed the animal could achieve was very high. Depending on the shape of the wind speed gradient assumed in our model, the animals reached maximum travel speeds of over 100 km/h even at realistic wind speeds of 5–10 m/s (Fig. 4), however, the average speeds reported using GPS for albatrosses and shearwaters were about 30–55 km/h (67, 69) (although, at wind speeds of around 20 m/s, an albatross was reported to travel at speeds of over 110 km/h for 9 hours (70)). There are 2 potential reasons for the discrepancy between this model and reality.

The first reason is that the actual shape of the wind speed gradient is likely to be more gradual than that assumed in the present study. It can be seen from our results in a sigmoidal wind gradient (Figures S1 and S2, Supplementary Material) that the gentler the wind speed variation (i.e. the larger the *δ*), the slower the maximum travel speed.

The second reason is that the upper limit of the bank angle change speed has not been considered. In dynamic soaring, there is a risk of wing failure due to the dynamic load on wings, which is higher than the static load during stable gliding as in thermal soaring. The risk of wing failure can be quantified by the ratio of the bending moment on the wing bones induced by lift to the stress that the bones can withstand. This ratio is called the “factor of safety” or “safety factor” (71–73). Animals should avoid situations with a low safety factor, limiting their bank angle change speed, and hence their flight speed. Few studies have quantified the safety factors of flying animals (72, 74), especially for extinct species, in part due to the need for detailed 3D geometrical data of bones and wings. However, Palmer's doctoral thesis (75) provides a plausible approach. Palmer quantified the safety factor along the wingspan of *Pteranodon* by applying the beam theory and vortex lattice method to the *Pteranodon* wing spar structural model. The computed safety factor was 2.5 at the first wing phalanx when Witton's mass estimates (9) were employed. Palmer concluded that this value is very low for a natural structure subjected to dynamic loading (73), suggesting that the dynamic load demand of dynamic soaring cannot be sustained by a *Pteranodon* wing. Applying Palmer's framework to other species would make for intriguing future research.

In view of the above 2 points, the dynamic soaring performance and the required wind conditions obtained from the present numerical calculation should not be taken literally by the values themselves. However, since all species were evaluated under the same assumptions, the relative values are meaningful indicators for the purpose of estimating soaring style by interspecies comparison, as was done in this study. A more refined analysis incorporating constraints on body rolling speed will be an interesting challenge in the future. For this purpose, actual measurements of bank angles in dynamic soaring birds and assessment of the wing bone structure and the lift force distributions along the wingspan in extant and extinct species will provide important information.

Understanding the soaring performance of extinct animals is an interdisciplinary issue. It is, therefore, essential to have a place for objective discussion between researchers with different backgrounds to bring their knowledge together, but such a platform seems to have been lacking in the past. In our approach, i.e. the physical model and the framework for comprehensively evaluating soaring performance, we have tried to clarify what assumptions were needed regarding the mechanics and the morphology of the animals. On the basis of our theoretical framework, it should, therefore, be easy for experts from various disciplines, including paleontologists, paleoclimatologists, engineers, and ornithologists, to examine the validity of the assumptions, examine new information, such as updated morphological data, and improve the model. We hope that our theoretical framework presented in this study will inspire researchers from various disciplines to work together to understand the soaring performance of extinct animals.

## Materials and Methods

### Quantification of soaring styles in previous studies

This section reviews previous studies on the soaring performance of extinct giant animals. In particular, we focus on which indices were quantitatively evaluated for each species.

#### Pelagornis sandersi

*P. sandersi* is predicted to be a dynamic soarer rather than a thermal soarer as its glide polar (and glide ratio that can be derived from its glide polar) is more similar to those of living dynamic soarers than those of living thermal soarers (1). However, this means the understanding of this species’ soaring style has been based on just 1 metric, that is, its glide ratio (Table 2). Hence, evaluating other metrics of this species could provide a more accurate estimate of the soaring style of this species. A previous study cautiously calculated the glide polars of *P. sandersi* for 24 combinations of estimates (Table 1) to deal with morphological uncertainty. Hence, we also employed these estimates in this study.

#### Argentavis magnificens

*A. magnificens* is expected to be a thermal soarer. A previous study reported that the thermal soaring performance and required wind conditions of this species were comparable to living thermal soaring species based on glide polars and circling envelopes (4). This result is consistent with the fact that an *Argentavis* specimen was found on the foothills and Pampas of Argentina, far from coastlines (4).

#### Pteranodon and Quetzalcoatlus

Although assessments of the soaring abilities of *Pteranodon* and *Quetzalcoatlus* have been a long-standing issue, there is still lack of a comprehensive understanding of their soaring style due to several uncertainties in the estimates of their morphology, especially because of the significant changes in weight estimates around 2010. Previously, it was estimated that *Pteranodon* had a wingspan of around 7 m and a body mass of 16 kg, while *Quetzalcoatlus* had a wingspan of around 11 m and a body mass of 50–70 kg. Based on these estimates, previous studies argued that they were adapted to thermal soaring (76, 77) and others argued that they could also employ dynamic soaring (33). Around 2010, however, several studies with different approaches suggested that pterosaurs were much heavier than previously expected (9, 14, 15) (Table 1). For example, Witton estimated that *Pteranodon* was 36.7 kg with a 6.0 m wingspan, and *Quetzalcoatlus* was 259 kg with a 9.6 m wingspan (9). Henderson estimated that *Pteranodon* was 18.6 kg with a 5.3 m wingspan, and *Quetzalcoatlus* was 544 kg with a 11.2 m wingspan (15). Witton and Habib argued that *Pteranodon* was a dynamic soarer and *Quetzalcoatlus* was a thermal soarer by comparing their morphology with those of extant soaring animals using PCA (9, 16, 41). Conversely, a recent study quantified the cost of transport and sinking speeds during gliding in pterosaur species and showed that azhdarchoid pterosaurs, including *Quetzalcoatlus*, had lower flight efficiency than the other pterosaurs (26). Despite these studies, the performance and wind requirements of dynamic and thermal soaring in these species have not been comprehensively quantified.

Furthermore, pterosaurs have a wing morphology that is completely different from that of birds and bats. Some studies reported that the wings of pterosaurs would have been associated with high-profile drag (34, 78). Palmer experimentally measured profile drag in a wind tunnel experiment using reconstructed pterosaur wings, and determined glide polars of *Pteranodon*-sized pterosaurs with various body mass estimates including the most recent heavy estimates (34). Palmer concluded that *Pteranodon* had the comparative thermal soaring ability to that of extant thermal soaring species, and that they adopted a slow flight speed. This conclusion was further reinforced by a subsequent work, which quantitatively showed that the pterosaur's wing membranes were not suited to fast flight (36).

In our analysis, we used the latest body mass estimates and drag coefficient estimates. For the drag coefficient, we used Palmer's experimental results (34). For body mass, there is still variation even among recent studies (9, 15). Therefore, for *Pteranodon*, we employed the 2 estimates by Henderson (15) and Witton (9). For *Quetzalcoatlus*, we only employed Witton's estimate (9), since Witton and Habib (16) pointed out a problem with applying Henderson's estimation method to giant azhdarchid pterosaurs.

### Models

The dynamics of soaring animals are described using the equations of motion (EOM). We first describe the EOM and parameters therein. We then describe the calculation procedure for dynamic soaring and thermal soaring, respectively.

#### Aerodynamic forces and parameters

We regard an animal as a point of mass. We employ the frame of reference $( {{\boldsymbol{i}}, {\boldsymbol{j}},{\boldsymbol{k}}} ) = ( {{{{\bf e}}_{{\rm{East}}}},{{{\bf e}}_{{\rm{North}}}},{{{\bf e}}_{{\rm{Up}}}}} )$ (44). Then, the dynamics of the animal's 3D position ${\boldsymbol{X}}( t ) = x{\boldsymbol{i}} + y{\boldsymbol{j}} + z{\boldsymbol{k}}$ and ground velocity ${\boldsymbol{V}}( t ) = {V_x}{\boldsymbol{i}} + {V_y}{\boldsymbol{j}} - {V_{{\rm{Sink}}}}{\boldsymbol{k}}$ are represented by the following EOMs:

(1) $$\begin{equation*}
{m\frac{{d{\boldsymbol{V}}}}{{dt}} = \boldsymbol{L }+ \boldsymbol{D} + m\boldsymbol{g}}
\end{equation*}
$$

and

(2) $$\begin{equation*}
m\frac{{d{\boldsymbol{X}}}}{{dt}} = {\boldsymbol{V}}.
\end{equation*}
$$

When an animal is soaring, 3 forces—gravitation (*m***g**), lift force (***L***), and drag force (***D***)—act on it. Gravitation *m**g*** is a product of the constant of gravitation (***g***) and mass of the bird (*m* kg), and its direction is toward the ground. The direction of the lift force***L*** is dorsal and perpendicular to the air velocity, ${{\boldsymbol{V}}_A}{\boldsymbol{}}( t ) = {\boldsymbol{V}}( t ) - {\boldsymbol{W}}( t )$, where ${\boldsymbol{W}}( t )$ represents the wind velocity. Drag force ***D*** is against the air velocity. For the analysis of dynamic soaring, we represent these EOMs in a different way by transforming the ground velocity to pitch $\gamma $, yaw $\psi $, bank angle $\phi $, and airspeed *V* ($\equiv | {{{\boldsymbol{V}}_A}} |$). The yaw angle $\psi $ is the angle between ${\boldsymbol{i}}$ and the projection of ${{\boldsymbol{V}}_A}$ to the **ij**-plane, and the pitch angle $\gamma $ is the angle between ${{\boldsymbol{V}}_A}$ and the **ij**-plane and is positive nose down. We assumed that wind blow is along the *y*-axis and wind only depends on the altitude of the animal, i.e. ${\boldsymbol{W}}( t ) = - W( z ){\boldsymbol{j}}$. Under this formulation the EOMs area represented by the following equations (44):

(3) $$\begin{equation*}
m{\rm{}}\frac{{dV}}{{dt}} = {\rm{}} - D + mg\sin \gamma + m\frac{{\partial W(z)}}{{\partial z}}\frac{{dz}}{{dt}}\cos \gamma \sin \psi ,
\end{equation*}
$$

(4) $$\begin{equation*}
mV\frac{{d\gamma }}{{dt}} = L\cos \phi - mg\cos \gamma + m\frac{{\partial W(z)}}{{\partial z}}\frac{{dz}}{{dt}}\sin \gamma \sin \psi ,
\end{equation*}
$$

(5) $$\begin{equation*}
mV\cos \gamma \frac{{d\psi }}{{dt}} = L\sin \phi + m\frac{{\partial W(z)}}{{\partial z}}\frac{{dz}}{{dt}}\cos \psi ,
\end{equation*}
$$

(6) $$\begin{equation*}
\frac{{dx}}{{dt}} = V\cos \gamma \cos \psi ,
\end{equation*}
$$

(7) $$\begin{equation*}
\frac{{dy}}{{dt}} = V\cos \gamma \sin \psi - W(z),
\end{equation*}
$$

and

(8) $$\begin{equation*}
\frac{{dz}}{{dt}} = {\rm{}} - V\sin \gamma ,
\end{equation*}
$$

where *W*(*z*) represents the wind gradient. A specific form is provided in the latter subsection. *L* represents the strength of the lift force, and *D* represents that of the drag force. The aerodynamic theory asserts that these values are

(9) $$\begin{equation*}
L= | {\boldsymbol{L}}|= \frac{1}{2}\rho {C_L}{S_{\rm{W}}}{V^2}
\end{equation*}
$$

and

(10) $$\begin{equation*}
D= | {\boldsymbol{D}}|= \frac{1}{2}\rho {C_{{\rm{Dpro}}}}{S_{\rm{W}}}{V^2} + \frac{1}{2}\rho {C_{{\rm{Dpar}}}}{S_{\rm{B}}}{V^2} + \frac{{\rho \left( {kC_{\rm{L}}^2} \right)}}{{2\pi {R_a}}}{S_{\rm{W}}}{V^2}.
\end{equation*}
$$

Here, *C*_L_ represents the lift coefficient, *S*_W_ represents the wing area, and *ρ* represents the air density and was set to *ρ* = 1.23 kg/m^3^ (26). This is the International Standard Atmosphere value for sea level at 15°C expressed as 3 significant digits (1). We employed this value because, based on a geochemical model of the carbon/sulfur cycle (79, 80), 20–23% of the air volume during the era of pterosaurs and giant extinct birds (approximately 70–6 Ma) was oxygen, which is almost the same as today (21%). We also tested the sensitivity of soaring performance to air density (See *Air density dependence of soaring performance*).

The drag is composed of 3 terms. The first term is the profile drag that stems from friction on the wing. *C*_Dpro_ is the profile drag coefficient. The *Flight* software developed for evaluating bird flight performance employs 0.014 as the default value for the *C_D_*_pro_ (21). This value is often used for birds (1) and even for pterosaurs (16). However, theory predicts that the profile drag coefficient varies with the lift coefficient following

(11) $$\begin{equation*}
{C_{{\rm{Dpro}}}} = {C_{{\rm{Dpro}},{\rm{min}}}}+ {k_{{\rm{pro}}}}{\left( {{C_L} - {C_{L,{D_{{\rm{pro}},{\rm{min}}}}}}} \right)^2},
\end{equation*}
$$

and experimental results support this prediction (34, 81–83). Hence, for birds, we employed *C*_Dpro,min_ = 0.019, *k*_pro_ = 0.030, and *C*_L,Dpro,min_ = 0.77, estimated from a jackdaw gliding in a wind tunnel (82). For pterosaurs, we obtained values from a wind tunnel experiment with reconstructed pterosaur wings (34). In the study (34), the reconstructed pterosaur wing span was approximately 6 m; however, we assumed that the result would not vary significantly with the size of *Quetzalcoatlus*. We quantified the position of data points in Fig. 3(b) of (34) using WebPlotdgitzer and fitted (Eq. 11) to the data points (see Figure S3, Supplementary Material). In (34), results of 2 types of wing sections are shown: faired wings and unfaired wings. For faired wings, we obtained *C*_Dpro,min_ = 0.050, *k*_pro_ = 0.11, and *C*_L,Dpro,min_ = 1.0 and, for unfaired wings, *C*_Dpro,min_ = 0.077, *k*_pro_ = 0.065, and *C*_L,Dpro,min_ = 0.89. Faired wings entail higher soaring efficiency compared to unfaired wings. For simplicity, we only show the results of faired wings in this study.

The second term is the parasite drag stemming from friction on the body, where the *C_Dpar_* is the parasite drag coefficient, and *S*_B_ is the body frontal area. We used the following recently recommended formula:

(12) $$\begin{equation*}
{C_{{\rm{Dpar}}}}{S_{\rm{B}}} = 0.01{S_{\rm{W}}},
\end{equation*}
$$

on the practical basis that neither *C*_Dpar_ nor *S*_B_ is exactly known (45).

The third term is the induced drag that stems from the lift force. *R_a_* represents the aspect ratio (*R*_a_ = *b*^2^/*S*_W_, where b is the wingspan). The *k* is the induced drag factor; we set *k* to 1.1, as in previous studies (1, 16, 21). The lift coefficient has a maximum value; for birds, we set *C*_L_ to $\le $ 1.8 (21). As the aerodynamic properties of pterosaurs can differ from those of birds, and the wind tunnel experiment indicated that *C*_L_ could reach more than 2.0 (34), we set the pterosaurs’ lift coefficient to $\le $ 2.0.

The remaining parameters in the EOMs are body mass (*m*), wingspan (*b*), and wing area (*S*_w_). For these morphological parameters of extant birds, we used values reported in previous studies (shown in Table 1).

The EOMs include variables that soaring animals can control, that is, bank angle $\phi $(t) and lift coefficient *C*_L_. Although these variables are time-dependent, for simplicity, we assumed that the animals keep their lift coefficients at a constant value. Hence, using a time series for bank angle, a constant value of *C*_L_, and values of parameters, the dynamics of the soaring animals were determined with EOM.

### Quantification of the dynamic soaring performance and the minimum required wind speed

#### Wind gradient models

We explored 2 types of wind gradients. The first was the logarithmic model represented as

[Logarithmic wind gradient model]

(13) $$\begin{equation*}
{W_{{\rm{Log}}}}(z) = \frac{{{W_{10}}}}{{\ln (10/{h_{{\rm{min}}}})}}\ln ( {z/{h_{{\rm{min}}}}}).
\end{equation*}
$$

This function is defined at *z* > *h*_min_. We set *h*_min_ to 0.03 [m], following a previous study (40). *W*_10_ is the wind speed at height *z* = 10 m. This model is deemed to be a good model of the average wind field in the first 20 m above the sea surface, assuming a flat sea surface, and has been a popular approach in dynamic soaring modeling. However, recent studies argued that the real sea surface is not flat, and wind separations in ocean waves may occur more often than expected (60). To describe wind-separation-like wind profiles, a sigmoidal model has been proposed (44, 84). We also employed the sigmoidal wind model with a minor change, represented as

[Sigmoidal wind gradient model]

(14) $$\begin{equation*}
{W_{{\rm{Sigmoid}}}}(z) = \frac{{{W_{{\rm{max}}}}}}{{1 + {{\rm{e}}^{ - \frac{{z - {h_{\rm{W}}}}}{\delta }}}}}.
\end{equation*}
$$

The *h*_w_ determines the height of wind separation, as shown in Fig. 3. In this study, we set *h*_w_ to 1, 3, and 5. The $\delta $ is the thickness parameter. The wind speed changes with height ($| {z - {h_w}} | \mathbin{{\buildrel<\over {\smash{\scriptstyle \sim}\vphantom{_x}}}} 3\delta $ m). In a previous study, the wind shear thickness was speculated as approximately 1.5–7 m. Here, we set $\delta $ to 3/6 with a steep wind change, and 7/6 with a gentler change (Fig. 3).

#### Formulation to numerical optimization

The numerical computation of dynamic soaring performance and minimum wind speed boiled down to the restricted optimization problem (85). That is, a mathematical problem to find the values of (i) a certain variable ***Y*** that maximizes (ii) an objective function *f*(***Y***), satisfying (iii) equalities ***h***(***Y***) = 0 and (iv) inequalities ***g***(***Y***) ≤ 0. In the following, we describe the variables, object functions, equalities, and inequalities for dynamic soaring.

##### Variables

The dynamics of dynamic soaring animals are described by the 3D position (*x*(t), *y*(t), *z*(t)), pitch angle $\gamma ( t )$, yaw angle $\psi ( t )$, airspeed *V*(*t*), bank angle $\phi ( t )$, lift coefficient *C*_L_, and the period of 1 dynamic soaring cycle $\tau $. Among these variables, 3D position, pitch, yaw, bank, and airspeed are functions of time *t* ($0 \le t \le \tau $). Optimization problems that include functions as variables are difficult to be directly solved. Therefore, we employed a collocation approach (43, 44). The collocation approach discretizes the variables in time, such as *X*(t) ($0 \le t \le \tau $) to variables *X_i_* = *X*((*i*-1)*N*/$\tau $) (*i* = 1, *N*), and converts the EOM to the equalities between those discretized variables. Hereafter, we use *X*_1:_*_N_* = {*X*_1_, *X*_2_, . . ., *X_N_*}. In this study, we set the number of discretization points to *N* = 51 in order to perform computations with reasonable accuracy within a reasonable amount of time. Accordingly, the variables of this optimization problem are position ${x_{1:N}},{y_{1:N}},{z_{1:N}}$, pitch angle${\gamma _{1:N}}$, yaw angle ${\psi _{1:N}}$, airspeed ${V_{1:N}}$, bank angle ${\phi _{1:N}}$, lift coefficient *C*_L_, and a period of 1 soaring cycle $\tau $. In addition, when computing the minimum wind speed required for sustainable dynamic soaring, *W*_10_ (log model) or *W*_max_ (sigmoid model) were also treated as variables. Hence, the total number of variables were 7 × 51 + 2 (+1 [when computing the minimum wind speed]) = 359 (or 360).

##### Object function

First, we computed (1) the minimum wind speed required for sustainable dynamic soaring for each wind gradient model. As the objective function to minimize, we set *W*_10_ for the logarithmic model and *W*_max_ for the sigmoidal model. Then, we computed (2) the maximum travel speed averaged over 1 dynamic soaring cycle by maximizing the object function to $\sqrt {x_N^2 + y_N^2} /\tau $. Finally, we computed (3) the maximum upwind speed averaged over 1 dynamic soaring cycle by maximizing the object function ${y_N}/\tau $. With respect to the maximum travel speed and maximum windward speed, we computed these values for different wind speeds, that is, from the minimum required wind speed of the species to the highest minimum required wind speed among the examined species (i.e. *Quetzalcoatlus*) + 2 m/s. In this wind speed range, the maximum travel speed reached an unrealistically high value and/or the optimization calculation did not converge for some species. Thus, we stopped the computation of the maximum travel speed at the wind speed where the maximum travel speed exceeded 40 m/s (144 km/h).

##### Equalities

The first equalities to be fulfilled for dynamic soaring animals are given in Eqs. 3–8. The collocation approach converts the EOM into the equalities between the variables listed in the above section. As the number of original EOM was 6 and the number of discretization was 51, the EOM were converted into 6 × 51 = 306 equalities (see (43, 44) for the specific representations of these equalities).

The second type of equalities to be fulfilled were periodic boundary conditions of dynamic soaring: at the beginning and end of 1 dynamic soaring cycle, the state of the animal (i.e. pitch, yaw, airspeed, bank, and height) is the same, represented as

(15) $$\begin{equation*}
{z_1} = {z_N},{\gamma _1} = {\gamma _N},{\psi _1} = {\psi _N},{\phi _1} = {\phi _N},{V_1} = {V_N}.
\end{equation*}
$$

##### Inequalities

First, we assumed that there was a maximum limit of physical load on the animal. This is because dynamic soaring entails dynamic maneuvering, which results in a corresponding acceleration. We employed the approach of a previous study (40) that restricted the load factor (*L*/*mg*) to less than 3,

(16) $$\begin{equation*}
\frac{L}{{mg}} \le 3.
\end{equation*}
$$

The second inequality was an important modification of the previous models. The height of the animal's wingtip above the sea surface (*z*_wing_) was calculated and represented as

(17) $$\begin{equation*}
{z_{{\rm{wing}}}} = z - \frac{b}{2}| {\sin \phi }|\cos \gamma \ge 0.
\end{equation*}
$$

Previous studies discarded the existence of the sea surface (44) or restricted birds to only flying higher than a given height (1.5 m) from the sea surface (40). However, the height an animal can fly depends on the wing length and the bank angle (e.g. with a shorter wing length and a lower bank angle, an animal can fly at a lower height). When dynamic soaring birds fly, they adjust their wingtips close to, but avoid touching, the sea surface (Fig. 3D). Dynamic soaring animals can exploit more flight energy when they pass through stronger wind speed gradients. As the wind speed difference is strong close to the sea surface, how close to the sea surface an animal can fly is crucial for dynamic soaring animals. Accordingly, long wings may restrict the minimum height at which the animal can fly and disturb efficient dynamic soaring. Hence, considering the effect of wings is crucial for evaluating dynamic soaring performances.

Third, we assumed that the height of the animal was higher than 0.5 m, that is

(18) $$\begin{equation*}
z \ge 0.5.
\end{equation*}
$$

The optimization problem described here is a restricted nonlinear optimization problem. We used the SQP method to solve the problem with the “fmincon” function in MATLAB^®萔^ Ver R2019a.

### Quantification of the thermal soaring performance and the required upward wind speed

For the computation of glide polars and circling envelopes, we followed a similar procedure to the *Flight* software, as described in (21), but modified some parameters and settings. Below, we outline the procedure and parameters employed in this study.

First, to compute the glide polars and circling envelopes, we needed to set rules regarding how gliding animals adjust their wingspan with respect to their airspeed. To achieve this, we used 2 wingspan adjustments: linear wingspan reduction and fixed wingspan (21). Although a fixed wingspan resulted in a better performance than linear wingspan reduction, we found no substantial differences in the results (see Fig. 5; Figures S4–S6 and Table S1, Supplementary Material). Hence, for birds, Fig. 5 shows the results of adopting linear wingspan reduction, which is *Flight's* default setting based on measurement of a jackdaw gliding in a wind tunnel (86). The default settings of *Flight* assume that wingspan, wing area, and thus aspect ratio linearly decreases with factor *β* = (*B*_stop_- *V*/*V_S_*)/(*B*_stop_- 1). Therefore, we replaced *b, S*_W_, and *R*_a_ with *βb, βS*_W_, and *βR*_a_, respectively. In this equation, *V_S_* is the stall speed, the airspeed of the animal at the highest lift coefficient (i.e. ${V_s} = \sqrt {( {2mg} )/(\rho {S_{\rm{W}}}{C_{{\rm{Lmax}}}}} $)); and *B*_stop_ is a constant that determines the degree of wing reduction. We set *B*_stop_ to 5, the default value in *Flight*. For pterosaurs, we show in Fig. 5 the results of a fixed wingspan. This is because, to the best of our knowledge, no study has yet shown a clear mechanism, whereby a membrane wing can be reduced in span and area without slacking the membrane. For reference, we also show results of the linear wingspan reduction, where *B*_stop_ was set to 6, in Figures S4–S6 (Supplementary Material) and Table S1 (Supplementary Material). This is a value employed in a previous study (16), although no quantitative evidence for this value was provided therein.

Then, the glide polars were derived from the EOM, setting bank angle to 0, assuming that the pitch angle was small enough ($\gamma \ll 1$) and considering the gliding animal was at kinematical equilibrium ($mg= \sqrt {{L^2} + {D^2}} \simeq L$, $\sin \gamma = D/L \simeq D/mg$). Sinking speed was represented as a function of airspeed *V* (21),

(19) $$\begin{equation*}
{V_{{\rm{Sink}}}} = \frac{\rho }{2}\left( {\frac{{{S_{\rm{W}}}}}{{mg}}} \right)\left( {\beta {C_{{\rm{Dpro}}}} + 0.01} \right){V^3} + \left( {\frac{{mg}}{{{S_{\rm{W}}}}}} \right)\left( {\frac{{2k}}{{\rho {R_{\rm{a}}}{\beta ^2}\pi }}} \right)\frac{1}{V}.
\end{equation*}
$$

Note that we used Eq. 12 (*C*_Dpar_*S*_B_ = 0.01*S*w; in this equation, *S*_W_ is not replaced with *βS*_W_) to derive the above equation. Eqs. 11, 19, and the equilibrium relation, ${C_L} = 2mg/( {\rho \beta {S_{\rm{W}}}{V^2}} )$, result in the following equation that gives a glide polar:

(20) $$\begin{equation*}
V_{\text{Sink}} = \frac{\rho}{2}\left(\frac{S_{\rm{W}}}{mg}\right)\Bigg\lbrace \beta \left[C_{\rm{D_{\text{pro}}},{\rm{min}}} + k_{\rm{pro}}\left(\frac{2mg}{\rho\beta S_{\rm{W}}V^{2}} - C_{L,{D_{\rm{pro}},{\rm{min}}}}\right)^2\right]\nonumber\\
+\, 0.01 \Bigg\rbrace V^{3} + \left(\frac{mg}{S_{\rm{W}}} \right)\left(\frac{2k}{\rho R_{\rm{a}}\beta ^2\pi} \right)\frac{1}{V}.
\end{equation*}
$$

The horizontal speed is $\sqrt {{V^2} - V_{{\rm{Sink}}}^2} $. Thus, the maximum glide ratio is the maximum value of $\sqrt {{V^2} - V_{{\rm{Sink}}}^2} /{V_{{\rm{Sink}}}}$.

Next, we examined the circling envelope. We considered a situation where an animal is gliding on a circular track of radius *r* with a bank angle of $\phi $ and the lift coefficient $C_L^*$. The circle radius and the sinking speed in that situation (*V*_Sink,Circle_) is given by the airspeed *V*($C_L^*$) and the sinking speed *V*_Sink_($C_L^*$) when the animal is gliding along a straight track with a lift coefficient $C_L^*$ as below (87).

(21) $$\begin{equation*}
r= \frac{{V{{\left( {C_L^*} \right)}^2}}}{{g\sin \phi }}
\end{equation*}
$$

and

(22) $$\begin{equation*}
{V_{{\rm{Sink}},{\rm{Circle}}}} = \frac{{{V_{{\rm{Sink}}}}\left( {C_L^*} \right)}}{{{{\left( {\cos \phi } \right)}^{3/2}}}},
\end{equation*}
$$

where $V{\rm{}}( {C_L^{\rm{*}}} ) = \sqrt {2mg/( {\rho \beta {S_w}C_L^{\rm{*}}} )} {\rm{}}$, and ${V_{{\rm{Sink}}}}( {C_L^{\rm{*}}} )$ is obtained by substituting $V( {C_L^{\rm{*}}} ){\rm{}}$ into Eq. 20.

There are 2 types of what we refer to as a "circling envelope” (21, 22, 88, 89). The circling envelope, proposed by Pennycuick (22), is an upper performance limit that an animal can achieve in the ascending phase of thermal soaring. The circling envelope was defined as a function that gives, for a circle radius *r*, a minimum sinking speed that an animal can achieve when gliding in a circle. Specifically, this is expressed as

(23) $$\begin{equation*}
\text{min}_{C_{L}^{*} \le {C_{L\text{max}}},0 \le \phi \lt \frac{\pi}{2}} {V_{{\rm{Sink}},{\rm{Circle}}}}( {V\left( {C_{L}^{*}}\right),\phi } )\,\,{\rm{with}}\,\,{\rm{the}}\,\,{\rm{constraint}}\nonumber\\
r= \frac{{V{{\left( {C_L^*} \right)}^2}}}{{g\sin \phi }}.
\end{equation*}
$$

At the limit where $r \to \infty $ (i.e. $\phi \to 0^\circ )$, this represents straight gliding flight with minimum sinking speed. The $C_L^*$ on the circling envelope varies with radius, and in the small radius region, it reaches to the maximum lift coefficient. This means that the animal is flying at a stall speed. Note that *r* should be larger than $V{( {{C_{L{\rm{max}}}}} )^2}/g$, which is the limit of $C_L^* = {C_{L{\rm{max}}}}$ and $\phi \to 90^\circ $. This minimum value is referred to as the limiting radius, i.e. the animal cannot achieve a circle radius smaller than this.

Furthermore, what Pennycuick newly called a circling envelope was a plot of Eqs. 21 and 22 as parametric equations with $\phi $ as the mediating variable, assuming $C_L^*$ to be constant (21). As for $C_L^*$, the lift coefficient minimizing the sinking speed during a straight glide was employed (21) (although there is a study that employed the maximum lift coefficient (90)). In this definition, at the limit where $\phi \to 0^\circ $, as in the former circling envelope, the animal is gliding in a straight line with a minimum sinking speed, but the limiting radius for $\phi \to 90^\circ $ is greater than the former. This is because the latter circling envelope assumes that the animal does not change its lift coefficient regardless of the circle radius. In other words, the former circling envelope shows an upper limit of the animal's capabilities, whereas the latter implicitly assumes that the animal is circling with some margin of safety that avoids the risk of stalling.

In recent years, the latter has often been used to determine the circling envelope (21, 51, 90). However, as the wings of pterosaurs are expected to have been able to achieve a higher maximum lift coefficient than that of birds (34), pterosaurs might have had a greater advantage than birds in increasing the lift coefficient when circling with a narrow radius. Therefore, to account for these differences, we evaluated both circling envelope definitions in this study. The 2 circling envelopes differed in the small circle radius, but there was no significant change in the relative performance relationship between the species. See Fig. 5 and Figure S5 (Supplementary Material) for results of the former and Figure S6 (Supplementary Material) for the latter results. The limiting radius, minimum sinking speed in straight gliding, maximum glide ratio, and horizontal speed at maximum glide ratio are shown in Table S1 (Supplementary Material) for the 2 wing reduction methods and 2 circling envelope definition. For ASK 14, the measured glide polar ( ${V_{{\rm{Sink}}}} = \frac{{10}}{V}+ 5.2 \times {10^{ - 5}}{V^3}$) and the circling envelope of the former definition setting maximum lift coefficient to 1.3, reported in a previous study (22), are presented.

### Air density dependence of soaring performance

To test the sensitivity to the air density, we also evaluated the soaring performance in high air density environments that pterosaurs and giant birds would have never experienced, i.e. at an air density of 1.2 times the current level (1.48 kg/m^3^). This air density corresponds to the value that would be obtained if the oxygen concentration increased to 30% and the amount of other components were equal to those in the present day (91). This air density is also expected to be higher than that during the eras of pterosaurs and giant extinct birds, as the concentration of oxygen reached 30%, approximately 300 Ma (Permian period), and had already decreased to a value comparable to modern day air in the eras of giant pterosaurs and giant extinct birds (70–6 Ma) (80).

We evaluated the dynamic soaring performances of *P. sandersi* and *Pteranodon* under this higher air density (see Figure S7, Supplementary Material) with wind gradient shown in Fig. 3. Although the higher air density resulted in a slightly lower minimum wind speed required for dynamic soaring, the qualitative results were similar to those obtained with the modern day air density (see Figure S7, Supplementary Material). We also evaluated the thermal soaring performance of *Quetzalcoatlus* under the higher air density (see Figure S8, Supplementary Material). Despite the high air density, the thermal soaring capability of *Quetzalcoatlus* remained low. Overall, our results were robust to air density uncertainty.

## Supplementary Material

pgac023_Supplemental_File

## Data Availability

All data needed to evaluate the conclusions in the paper are present in the paper and the Supplementary Materials.
