# Supplementary material for: How did extinct giant birds and pterosaurs fly? A comprehensive modeling approach to evaluate soaring performance
Source: PNAS Nexus. 2022 Mar 10;1(1):pgac023. doi: 10.1093/pnasnexus/pgac023 (PMC9802081; doi:10.1093/pnasnexus/pgac023)
Supplement: pgac023_Supplemental_File [file pgac023_supplemental_file.docx]

**Supplementary Information for**

How did extinct giant birds and pterosaurs fly? A comprehensive modeling approach to evaluate soaring performance

Yusuke Goto, Ken Yoda, Henri Weimerskirch, Katsufumi Sato

Yusuke Goto

e-mail: [goto924@gmail.com](mailto:goto924@gmail.com)

**This PDF file includes:**

Figures S1 to S8

Table S1

Supplementary text

**Figure S1:** Dynamic soaring performance of a sigmoidal wind model

with a wind shear thickness of 7 m (δ = 7/6) and a wind shear height of 1, 3, and 5 m.

**Figure S2:** Dynamic soaring performance of a sigmoidal

wind model with a wind shear thickness of 3 ­m (δ = 3/6) and a wind shear height of 1, 3, and 5 m.

**Figure S3:** Dependence of the profile drag coefficient on the lift coefficient for pterosaurs (based on Palmer

2011).

**Figure S4:** Glide polar with linear wingspan reduction and a fixed wingspan.

**Figure S5:** Circling envelope with the lowest sinking speed at circling glide.

**Figure S6:** Circling envelope with the lift coefficient at minimum sinking speed in straight glide.

**Figure S7:** Dynamic soaring performance of *Peragornis sanderisi* and *Pteranodon* at 1.2 times higher air

density.

**Figure S8:** Thermal soaring performance of *Quetzalcoatlus* at 1.2 times higher air density.

**Table S1:** Soaring performance with different wingspan adjustment and definition of the circling envelope.

**Figure S1 Dynamic soaring performance of a sigmoidal wind model with a wind shear thickness of 7 m (**δ **= 7/6) and a wind shear height of 1, 3, and 5 m.**

**Figure S2 Dynamic soaring performance of a sigmoidal wind model with a wind shear thickness of 3 ­m (**δ **= 3/6) and a wind shear height of 1, 3, and 5 m.**

**Figure S3 Dependence of the profile drag coefficient on the lift coefficient for pterosaurs (based on Palmer 2011).**

The data points are the experimental results presented in Fig. 3 of Palmer (2011), where the blue points represent unfaired wings and the red points represent faired wings. The red and blue lines are quadratic functions fitted to the results of the faired and unfaired wings, respectively. The unfilled blue points were excluded when fitting of the quadratic function. The orange line is the result of a jackdaw experiment reported in KleinHeerenbrink et al. (2016).

­

**Figure S4 Glide polar with linear wingspan reduction and a fixed wingspan.** Solid lines represent glide polars with a fixed wingspan, and dashed lines represent those with linear wing reduction. The points at the best gliding ratio are represented by filled circles for a fixed wingspan and stars for linear wing reduction. For the linear wing reduction, B_stop_ = 5 was employed for birds and B_stop_ = 6 for pterosaurs.

**Figure S5 Circling envelope with the minimum sinking speed at circling glide.** Solid lines represent fixed wingspan, and dashed lines represent linear wingspan reduction.

**Figure S6 Circling envelope with the lift coefficient at minimum sinking speed in straight glide.** Solid lines represent fixed wingspan, and dashed lines represent linear wingspan reduction.

**Figure S7** **Dynamic soaring performance of *Peragornis sanderisi* at 1.2 times higher air density.** This figure is based on Fig. 4 with the addition of dotted lines representing the performance of *Peragornis sandersi* and *Pteranodon* under an air density 1.2 times that of the current era.

**Figure S8 Thermal soaring performance of *Quetzalcoatlus* at 1.2 times higher air density.** This figure is based on Fig. 5 with the addition of a green dotted line representing the performance of *Quetzalcoatlus* under an air density 1.2 times that of the current era. The lines of *Pelagornis sandersi* has been removed for visibility.

**Table S1 Soaring performance with different wingspan adjustment and definition of the circling envelope.**
